# Comprehensive circular RNA expression profiling constructs a ceRNA network and identifies hsa_circ_0000673 as a novel oncogene in distal cholangiocarcinoma

**DOI:** 10.18632/aging.104099

**Published:** 2020-11-18

**Authors:** Xin Zhao, Xinxue Zhang, Zhigang Zhang, Zhe Liu, Jiqiao Zhu, Shaocheng Lyu, Lixin Li, Ren Lang, Qiang He

**Affiliations:** 1 Department of Hepatobiliary Surgery, Beijing Chao-Yang Hospital Affiliated with Capital Medical University, Beijing, China; 2 School of Information Management and Statistics, Hubei University of Economics, Wuhan, Hubei Province, China

**Keywords:** distal cholangiocarcinoma, circular RNA, competing endogenous RNAs, microarray, hsa_circ_0000673

## Abstract

Circular RNAs (circRNAs) play an important role in cholangiocarcinoma (CCA) development; however, the expression and functions of circRNAs in distal CCA (dCCA) remain unknown. Herein, we explored the expression profile of circRNAs in six paired dCCA tumor and adjacent normal tissue samples using microarray. A total of 171 differentially expressed (DE) circRNAs were identified in dCCA tissues. Host genes of DE circRNAs were enriched in the cellular cytoskeleton and adheren junction. Bioinformatics analyses were used to establish a circRNA-microRNA-mRNA network for dCCA. Protein-protein interaction networks were constructed, and five hub genes were associated with the regulation of the cell cycle based on gene set enrichment analyses. Five DE circRNAs were validated with qRT-PCR in 40 pairs of dCCA tissues, and hsa_circ_0000673 showed promising diagnostic performance in distinguishing dCCA from normal tissues (AUC = 0.85, *p <* 0.01). Overexpression of hsa_circ_0000673 was associated with tumor invasion (*p* = 0.001), poor differentiation (*p* = 0.041), and residual tumor (*p* = 0.044). *In vitro* experiments indicated that inhibition of hsa_circ_0000673 suppressed the proliferation, migration, and invasion of CCA cells. This research provided a landscape of dysregulated circRNAs in dCCA and identified hsa_circ_0000673 as a potential biomarker and therapeutic target for dCCA.

## INTRODUCTION

Distal cholangiocarcinoma (dCCA) originates from epithelial cells of the common bile duct and accounts for approximately 20%-30% of all cholangiocarcinoma (CCA) cases [1]. The five-year overall survival of dCCA patients remains dismal, ranging from 11%-48% [2–4]. Most patients are diagnosed in the progressive stage and lost the opportunity for surgical resection. This delayed diagnosis is mainly due to a lack of specific biomarkers and a limited understanding of the molecular mechanisms regulating the oncogenesis of dCCA.

Recently, circular (circ)RNA, a type of non-coding RNA, has become recognized as a functional molecule in human diseases [5]. Unlike linear RNA, circRNA contains covalently closed loops without 3′ and 5′ ends, making it more stable and resistant to degradation. CircRNA has been shown to play essential regulatory roles in multiple biological processes, including transcriptional splicing, protein-protein interactions (PPIs), ribosomal RNA processing, and micro (mi)RNAs sponging [6]. Accumulating studies have demonstrated that circRNAs are differentially expressed (DE) in human cancers. For instance, hsa_circ_0007142 is upregulated and promotes cell proliferation in colorectal cancer [7]; in non-small cell lung cancer cell lines, hsa_circ_100146 is highly expressed [8]; in CCA cells, hsa_circ_0001649 is downregulated and exhibits tumor-suppressive activity [9]; and circRNA Cdr1as is upregulated in CCA cells and associated with poor prognosis [10].

A growing number of studies have revealed that circRNAs function as a sponge of miRNAs and inhibit their activities. Examples include the upregulation of hsa_circ_0005230, which facilitates cell growth and metastasis in CCA via sponging of miR-1238 and miR-1299 [11]. In hepatocellular carcinoma (HCC), hsa_circ_100338 activates the mammalian target of rapamycin signaling pathway by absorbing miR-141-3p [12]. In gastric cancer, hsa_circ_100269 inhibits cell proliferation by sponging miR-630 [13]. Therefore, at the post-transcriptional level, circRNAs regulate protein-coding mRNAs by competing for miRNAs; they are known as competing endogenous RNAs (ceRNAs) in this context [14]. Early studies demonstrated that circRNAs could be detected in body fluids, serum, plasma, and tissue samples [15, 16]. Consequently, they have the real potential of being promising molecular biomarkers and therapeutic targets in human diseases.

**Figure 1:**
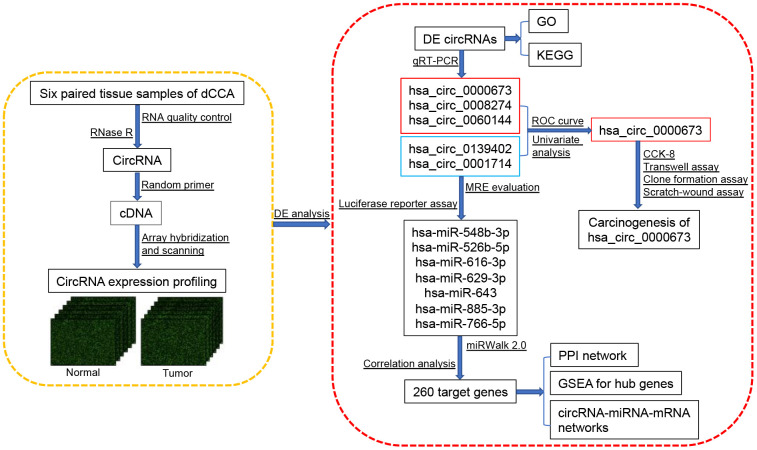
**Flowchart of the current study.** Abbreviations: dCCA: distal cholangiocarcinoma; circRNA: circular RNA; DE circRNAs: differentially expressed circular RNAs; GO: Gene Ontology; KEGG: Kyoto Encyclopedia of Gene and Genomes; qRT-PCR: quantitative real-time polymerase chain reaction; ROC curve: receiver operating characteristic curve; CCK-8: cell counting kit-8; MRE: microRNA response element; miRNA: microRNA; PPI: protein-protein interaction; GSEA: gene set enrichment analysis.

However, there has been little systematic evaluation of circRNAs in dCCA, and the function of dysregulated circRNAs remains to be further investigated. In this study, we constructed a comprehensive circRNA expression profile by microarray, performed DE analyses, identified a novel circRNA biomarker, and established a circRNA-miRNA-mRNA network to understand the possible functions of circRNAs in dCCA (Figure 1).

## RESULTS

### Identification of dysregulated circular RNAs in distal cholangiocarcinoma

**Figure 2:**
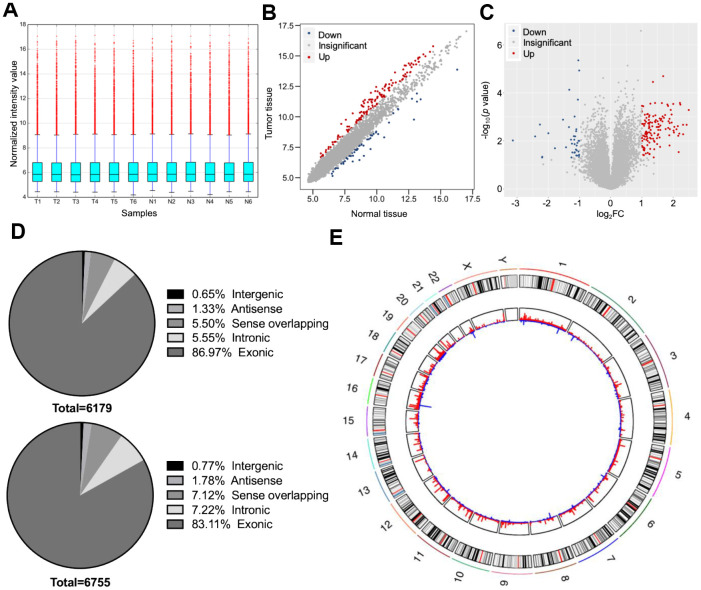
**Overall characteristics of circular (circ)RNA expression profiling of distal cholangiocarcinoma (dCCA).** (**A**) The distributions of circRNA expression values after quantile normalization and log2 processing. (T: dCCA tissues; N: paired adjacent normal tissues). (**B**) The scatter plot describing the difference of circRNA levels between dCCA and normal tissues. The red and blue dots indicate more than 1.5-fold change (FC) in dCCA and normal tissues, respectively. (**C**) The volcano plot of differentially expressed circRNAs (|log2FC| > 1 and p < 0.05). The red and blue dots represent up- and downregulated circRNAs in tumor. (**D**) The classifications of circRNAs (up: upregulated circRNAs; down: downregulated circRNAs). (**E**) The circle diagram describes the location of circRNAs on chromosomes. The red and blue lines represent the up- and downregulated circRNAs in tumor tissues. The segment length indicates the FC value.

**Figure 3:**
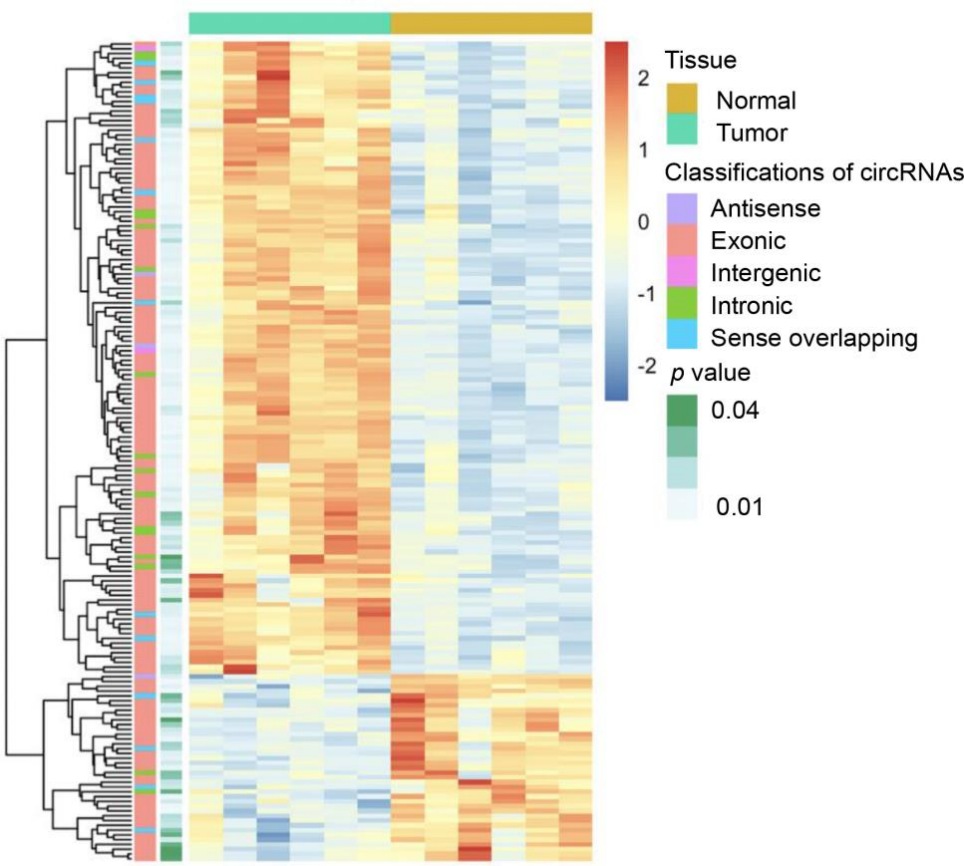
**Heatmap of differentially expressed circular (circ)RNAs in distal cholangiocarcinoma.** In total, 132 up- and 39 downregulated circRNAs were identified with microarray (|log2(fold change)|> 1 and p < 0.05). The green and yellow bars on the top represent tumor and normal tissues. In the middle, the color of boxes changes from red to blue, indicating high to low-expression of circRNAs. The first and second columns on the left represent classifications of circRNAs and the p-value of the differential analysis.

Six dCCA patients were enrolled in this study. The clinicopathological characteristics of the patients are summarized in Table 1. Microarray profiling analyses detected a total of 12,934 circRNAs. The distribution of the current dataset was appropriate (Figure 2A). A total of 132 up- and 39 downregulated circRNAs (|log_**2**_(fold change) (log_**2**_FC)| > 1 and *p* < 0.05) were identified and visualized with scatter and volcano plots (Figure 2B, 2C). The results indicated that more than 80% of the circRNAs originated from exons of host genes (Figure 2D). Upregulated circRNAs were mainly distributed in chromosomes 1 and 16, whereas downregulated circRNAs were enriched in chromosomes 17 and 19 (Figure 2E). In hierarchical clustering analyses, 171 DE circRNAs were distinguishable between dCCA and adjacent normal samples (Figure 3). The majority of DE circRNAs were also derived from exons. The characteristics of the top 10 dysregulated circRNAs are listed in Supplementary Table 1. All the microarray datasets were deposited in the Gene Expression Omnibus (GEO) site (GEO accession number: GSE148561).

**Table 1:** Clinicopathological characteristics of six patients with distal cholangiocarcinoma.

**No.**	**Gender**	**Age**	**Histological differentiation**	**T**	**N**	**M**	**TNM stage^a^**	**CA19-9 (U/mL)**	**Positive lymph nodes**	**Total lymph nodes**	**Perineuronal invasion**
1	Male	70	Moderate	T2	N0	M0	IIA	40.30	0	37	No
2	Male	62	Moderate-poor	T2	N0	M0	IIA	26.47	0	14	Yes
3	Male	64	Moderate	T3	N1	M1	IV	643.47	1	15	Yes
4	Male	69	Moderate	T3	N0	M0	IIB	74.04	0	21	Yes
5	Male	53	Moderate	T3	N0	M0	IIB	9.16	0	14	Yes
6	Female	56	Poor	T3	N1	M0	IIB	177.21	1	29	Yes

^a^TNM stage is based on the 8^th^ edition of the American Joint Committee on Cancer.

CA 19-9, carbohydrate antigen 19-9.

### Functions of differentially expressed circular RNAs

**Figure 4:**
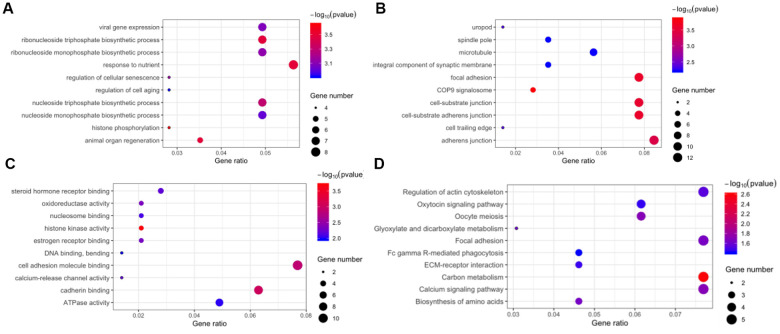
**Function annotation and pathway enrichment for the host genes of differentially expressed circular RNAs.** Gene Ontology, including biological process (**A**), cellular component (**B**), and molecular function (**C**). (**D**) Kyoto Encyclopedia of Gene and Genomes pathway enrichment.

The host genes of the 171 DE circRNAs were selected for Gene Ontology (GO) analyses. The results indicated that the term “response to nutrient” was most enriched in the biological process category (Figure 4A), and “adherens junction” was the top enriched term in the cellular component category (Figure 4B). The most significant number of genes in the molecular functions category were in “cell adhesion molecule binding” (Figure 4C). In Kyoto Encyclopedia of Gene and Genomes (KEGG) pathway enrichment analysis, five genes were associated with regulation of “actin cytoskeleton,” “focal adhesion,” “carbon metabolism,” and “calcium signaling pathway” (Figure 4D).

### Validation of differentially expressed circular RNAs

**Figure 5:**
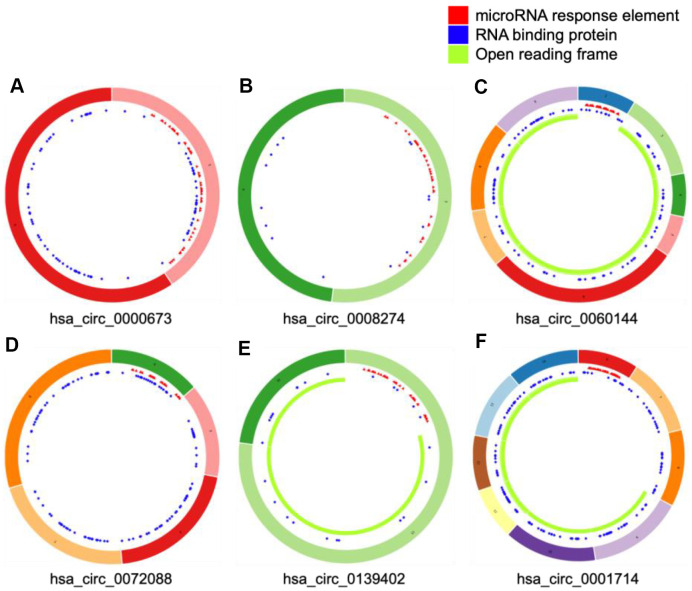
**Structural patterns of six differentially expressed circular (circ)RNAs.** The features of four upregulated circRNAs, including hsa_circ_0000673, hsa_circ_0008274, hsa_circ_0060144, and hsa_circ_0072088 (**A**–**D**), and two downregulated circRNAs, including hsa_circ_0139402 and hsa_circ_0001714 (**E** and **F**), were visualized by Cancer-Specific CircRNAs Database.

**Figure 6:**
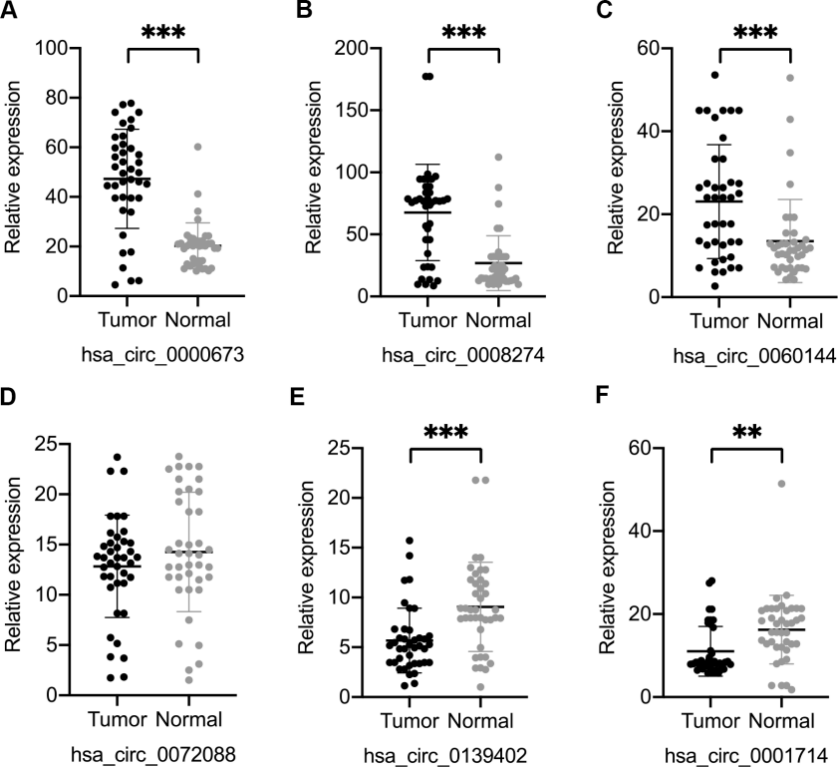
**Validation of six differentially expressed circular (circ)RNAs by qRT-PCR.** Three circRNAs, including hsa_circ_0000673 (**A**), hsa_circ_0008274 (**B**), and hsa_circ_0060144 (**C**) were significantly upregulated, and two circRNAs, including hsa_circ_0139402 (**E**) and hsa_circ_0001714 (**F**) were significantly downregulated in tumor tissues. (**D**) The level of hsa_circ_0072088 was not different between tumor and normal tissues. **p < 0.01, ***p < 0.001.

We selected four significantly upregulated circRNAs, including hsa_circ_0060144, hsa_circ_0008274, hsa_circ_0000673, and hsa_circ_0072088, and two significantly downregulated circRNAs, including hsa_circ_0139402 and hsa_circ_0001714 for quantitative real-time polymerase chain reaction (qRT-PCR) validation. The relative intensity values measured with microarray are shown in Supplementary Figure 1, and the structures of the six circRNAs were visualized using the Cancer-Specific CircRNA Database (CSCD) in Figure 5. The qRT-PCR results revealed that the expression levels of hsa_circ_0060144, hsa_circ_0008274, and hsa_circ_0000673 were significantly upregulated (Figure 6A–6C), and the expression levels of hsa_circ_0139402 and hsa_circ_0001714 were significantly downregulated (Figure 6E, 6F). However, the expression of hsa_circ_0072088 did not differ between tumor and healthy tissues (Figure 6D). Thus, the five verified DE circRNAs were selected to perform further bioinformatics analyses.

### A circRNA-miRNA-mRNA interacting network

Prior studies have shown that circRNAs act as miRNA sponge molecules and increase the expression of downstream genes. Hence, we predicted nine target miRNAs of the five verified DE circRNAs using CircInteractome and CSCD databases. The results of luciferase reporter assay validated the bindings between five circRNAs and seven miRNAs, including hsa_circ_0000673/miR-548b-3p, hsa_circ_0008274/miR-526b-5p, hsa_circ_0060144/ (miR-616-3p, miR-629-3p, and miR-643), hsa_circ_0139402/miR-885-3p, and hsa_circ_0001714/miR-766-5p (Supplementary Figure 2).

Using the DIANA-miRPath platform, we explored and visualized the KEGG pathways associated with the above seven miRNAs. The results showed that these miRNAs were mostly enriched in cancer-related pathways, basal cell carcinoma, TGF-beta, and Wnt signaling pathways (Supplementary Figure 3).

**Figure 7:**
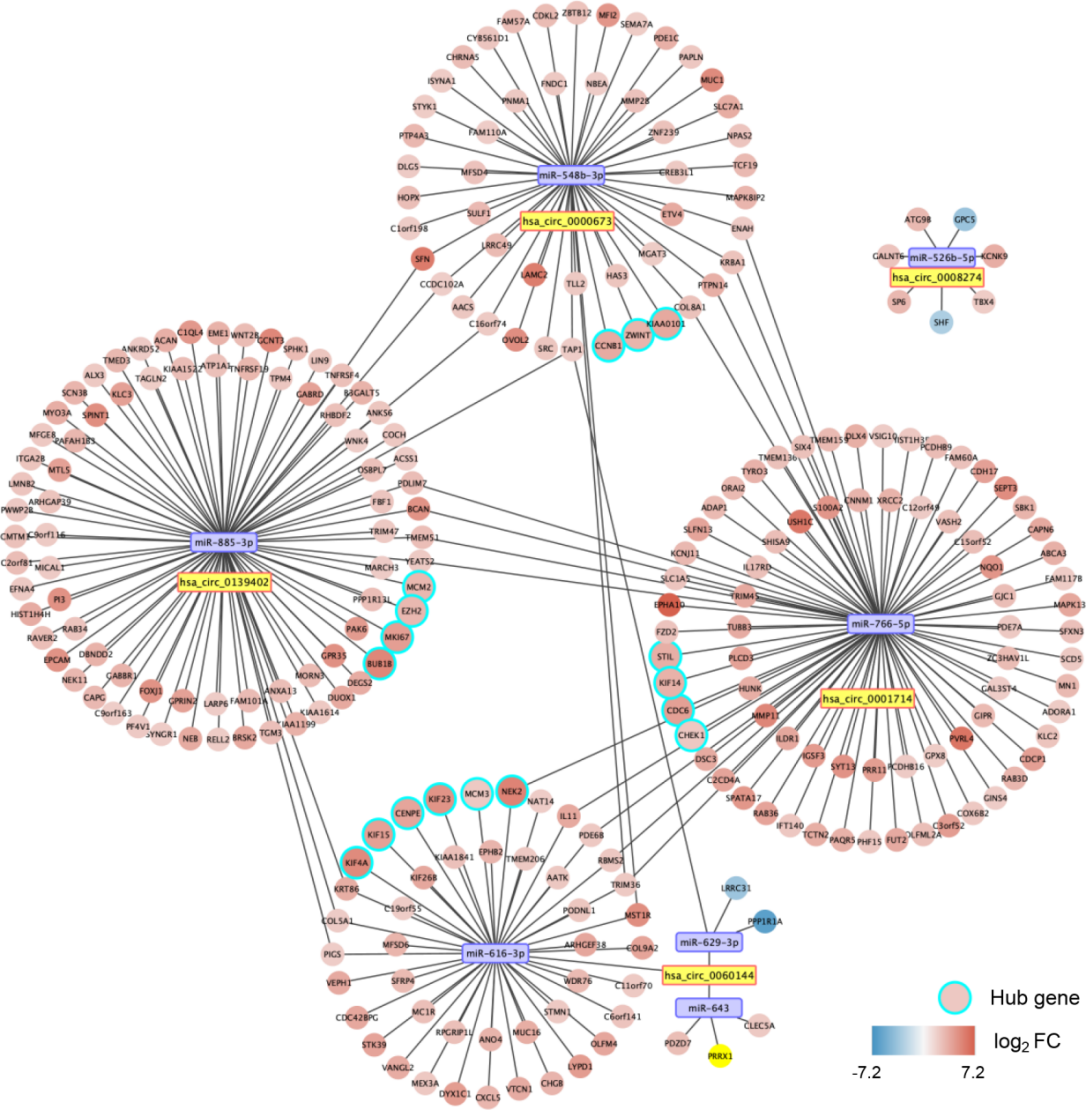
**A circular (circ)RNA-micro(mi)RNA-mRNA interacting network in dCCA.** The network contains five dysregulated circRNAs, seven miRNAs, 260 mRNAs, and 17 hub genes. The node color changes from blue to red, representing the fold change of mRNA expression value in the cholangiocarcinoma cohort of The Cancer Genome Atlas (tumor versus normal).

With miRWalk 2.0, we predicted the mRNAs with binding sites corresponding to the seven miRNAs. After that, we performed correlation analyses between miRNA and mRNA with the CCA cohort of The Cancer Genome Atlas (TCGA). A total of 260 significantly negatively correlated mRNAs were regarded as the potential target genes of the seven miRNAs. Thus, a circRNA-miRNA-mRNA interacting network was constructed, including five circRNAs, seven miRNAs, and 260 mRNAs (Figure 7).

### Gene set enrichment analysis of hub genes

A PPI network for the 260 target genes was constructed based on the Search Tool for the Retrieval of Interacting Genes (STRING) database and visualized with Cytoscape software (Supplementary Figure 4). Using the Molecular Complex Detection (MCODE) approach, we screened out 17 hub genes (Supplementary Figure 4). Thus, we established a potential critical ceRNA network for dCCA, including four DE circRNAs, four miRNAs, and 17 hub genes (Figure 7).

**Figure 8:**
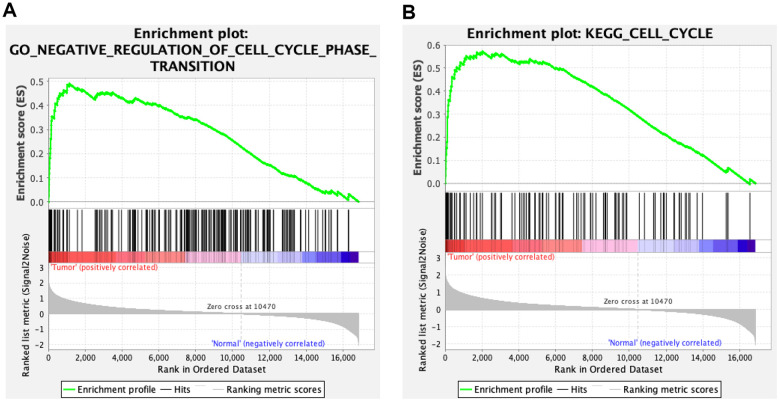
**Gene set enrichment analysis (GSEA) for cholangiocarcinoma (CCA).** Five hub genes of CCA, including BUB1B, CCNB1, CDC6, CHEK1, and MCM2 were enriched in the cell cycle based on biological process (**A**) and Kyoto Encyclopedia of Genes and Genomes analyses (**B**).

We validated the expression of the 17 hub genes with GSE32879 containing 23 CCA and seven normal samples. The results showed that 15 hub genes, including *CCNB1*, *KIF23*, *KIF4A*, *ZWINT*, *CENPE*, *KIF15*, *MCM3*, *NEK2*, *BUB1B*, *EZH2*, *MCM2*, *MIKI67*, *CHEK1*, *KIF14*, and *PCLAF* were significantly upregulated in tumor tissues (Supplementary Figure 5). Gene set enrichment analysis (GSEA) was performed based on the mRNA expression of the CCA cohort of the TCGA. As shown in Figure 8, compared with healthy tissue samples, *BUB1B*, *CCNB1*, *CDC6*, *CHEK1*, and *MCM2* were enriched in “negative regulation of cell cycle transition” in GO term of biological process and associated with “cell cycle” pathways in KEGG analyses. In summary, these results elucidate that the central circRNA-miRNA-mRNA network possibly regulates cell proliferation in dCCA.

### Association between hsa_circ_0000673 and dCCA progression

The results of the receiver operating characteristic (ROC) curve indicated that, in distinguishing tumor from healthy tissue samples, hsa_circ_0000673 had the highest area under the curve (AUC) value among the five circRNAs (Supplementary Figure 6). As shown in Table 2, overexpression of hsa_circ_0000673 was significantly associated with tumor invasion (*p* = 0.001), poor differentiation (*p* = 0.041), and residual tumor (*p* = 0.044). These results indicated that hsa_circ_0000673 was associated with malignant phenotypes of dCCA.

**Table 2:** Association between hsa_circ_0000673 level and clinicopathological parameters of distal cholangiocarcinoma patients.

**Clinicopathological parameters**	**hsa_circ_0000673^a^**		***p* value**
	**Low(n = 20)**	**High(n = 20)**	
Age (years)	66	69	0.436
Gender			0.731
Male	15	13	
Female	5	7	
Pathological differentiation			0.041*
Moderate and high	17	10	
Poor	3	10	
Numbers of positive lymph node	13.5±11.3	12.8±10.7	0.842
Vascular invasion			
Negative	17	18	1.000
Positive	3	2	
TNM stage^b^			1.000
I and II	8	9	
III and IV	12	11	
Primary tumor			0.001**
T1 and T2	15	4	
T3 and T4	5	16	
Reginal lymph nodes invasion			1.000
N0 and N1	8	9	
N2	12	11	
Distant metastases			0.4872
Negative	20	18	
Positive	0	2	
Resection			0.044*
R0	19	13	
R1 and R2	1	7	

*p < 0.05; **p < 0.01.

^a^Using median expression value of hsa_circ_0000673 as a cut-off.

^b^TNM stage is based on the 8^th^ edition of the American Joint Committee on Cancer.

R0: a negative resection margin; R1: a resection margin with microscopic residual tumor; R2: a resection margin with macroscopic residual tumor.

### Inhibition of hsa_circ_0000673 suppresses CCA cell proliferation, migration, and invasion *in vitro*

**Figure 9:**
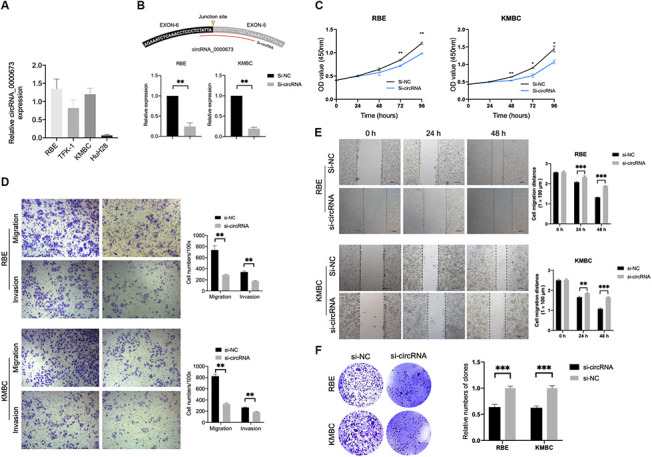
**Inhibition of hsa_circ_0000673 suppresses cholangiocarcinoma (CCA) cell proliferation, migration, and invasion.** (**A**) Relative expression of hsa_circ_0000673 in four CCA cell lines. (**B**) Specific small interfering RNA (siRNA) inhibits the expression of hsa_circ_000067 in CCA cells. Cell counting kit-8 assays (**C**), transwell migration and invasion assays (**D**), scratch-wound assays (**E**), and colony formation assays (**F**) of CCA cells transfected with negative control and si-hsa_circ_0000673. *p < 0.05, **p < 0.01, ***p < 0.001.

In order to investigate the biological functions of hsa_circ_0000673, we first measured its expression level in four CCA cell lines. As shown in Figure 9A, RBE showed the highest expression, and HuH28 exhibited the lowest expression. Therefore, we chose RBE and KMBC for the silencing of hsa_circ_0000673. The small interfering RNA (siRNA) targeting the back-splice junction sites of hsa_circ_0000673 was designed and shown to decrease the expression level significantly (Figure 9B). A cell counting kit-8 (CCK-8) assay showed that inhibition of hsa_circ_0000673 significantly suppressed cell proliferation in both RBE and KMBC cells (Figure 9C). In transwell assays, the silencing of hsa_circ_0000673 significantly inhibited the migration and invasion abilities of RBE and KMBC cells (Figure 9D). A scratch-wound assay revealed that the migration abilities of RBE and KMBC cells were significantly inhibited following treatment with siRNA (Figure 9E). Moreover, in colony formation assays, siRNA significantly decreased the colony-forming ability of RBE and KMBC cells (Figure 9F). These *in vitro* experiments collectively suggested that inhibition of hsa_circ_0000673 could suppress the proliferation, migration, and invasion of CCA cells.

## DISCUSSION

Cholangiocarcinoma, an aggressive tumor, is classified as intrahepatic CCA, perihilar CCA, or distal CCA based on anatomical location. Each subtype has distinct clinicopathological characteristics and therapeutic strategies [17]. To our knowledge, this is the first study conducting circRNA expression profiling of dCCA and revealing 132 up- and 39 downregulated circRNAs. The functions of DE circRNAs were illustrated with GO and KEGG analyses. Three up- and two downregulated circRNAs were validated with qRT-PCR. A circRNA-miRNA-mRNA network was constructed, including five circRNAs, seven miRNAs, and 260 mRNAs in dCCA. Finally, hsa_circ_0000673 was identified as a potential biomarker for dCCA, and inhibition of this circRNA suppressed proliferation, migration, and invasion of CCA cells *in vitro*.

In the GO term of biological process, “response to nutrient” and “ribonucleoside triphosphate biosynthetic process” were most enriched for DE circRNAs, which indicated that dysfunction of nutrient and energy metabolism might promote oncogenesis of dCCA. In cellular component and molecular functions, the term “adherens junction” was mainly enriched, which was in agreement with a previous study [18]. In KEGG analysis, “regulations of cytoskeleton” were significantly enriched, supporting the correlation between DE circRNAs and cellular migration [11].

In the current study, six circRNAs were selected for qRT-PCR according to the following criteria: first, the mature sequence length of circRNA was between 200–4000 bp; second, circRNAs better contained many binding sites with miRNA and RNA binding proteins; third, the top 20 dysregulated circRNAs were preferred. By doing this, the candidate circRNAs were more likely to be functional and suitable for subsequent experiments. Among the five verified circRNAs, hsa_circ_0000673 was identified as a biomarker for dCCA diagnosis with optimal efficiency (AUC = 0.85). It was associated with features of malignancy such as tumor invasion, poor histological stage, and residual tumor. The results of *in vitro* studies indicated that inhibition of hsa_circ_0000673 suppressed the migration and invasion abilities of CCA cells. Therefore, our results demonstrated that hsa_circ_0000673 could be an oncogene in dCCA and could serve as a potential biomarker and therapeutic target for dCCA diagnosis and treatment.

Hsa_circ_0000673 is derived from the fifth and sixth exons of RSL1D1 containing 251 bp. According to our study, hsa_circ_0000673 was verified to bind to miR-548b-3p, which may target *CCNB1*. In the CCA cohort of TCGA, *CCNB1* was significantly overexpressed in tumor tissues. Hence, hsa_circ_0000673 possibly promotes dCCA progression via miR-548b-3p/CCNB1. However, the potential regulatory mechanism is needed for further verification.

Our results identified five miRNAs that bind with the three upregulated circRNAs, and among them, miR-548b-3p, miR-526b-5p, and miR-616-3p are linked to pathways in cancer according to miRPath analyses. Studies revealed the antitumor activities of these miRNAs. For example, in glioma, miR-526b-5p and miR-548b-3p acted as tumor suppressors by targeting WEE1 [19] and MTA2 [20], respectively. In lung cancer, miR-548b-3p was downregulated in tumor specimens and induced cell apoptosis via the PI3K/AKT signaling pathway [21]. Moreover, results of KEGG analyses indicated that miR-629-3p and miR-643 were associated with the cell cycle pathway that promoted tumor progression. In pancreatic and gastric cancers, miR-629 acts as an oncogene, promoting cell proliferation and inhibiting apoptosis by targeting *FOXO3* [22, 23]. In osteosarcoma, miR-643 was revealed to suppress tumorigenesis by *ZEB1* [24].

On the other hand, miR-766-5p and miR-885-3p bind to the two downregulated circRNAs, and they were enriched in multiple cancer-related pathways. For example, in HCC, miR-885-3p and miR-766 played a role in tumor progression by targeting *TIGAR* and *NR3C2*, respectively [25, 26]. Likewise, these two miRNAs increased cell proliferation and migration by targeting *SOCS5* and *SCAI* in colorectal cancer [27, 28].

In the PPI network, 17 hub genes corresponding to four DE circRNAs and four miRNAs were identified. Interestingly, most of the hub genes were related to carcinogenesis. For example, the protein encoded by *CCNB1* plays a crucial role in regulating the G2/M transition. In gastric, breast, and colorectal cancer cells, inhibition of *CCNB1* induced G2/M phase cell cycle arrest and promoted apoptosis [29, 30]. Furthermore, *CHEK1* [31], *BUB1B* [32], and *MCM2* [33] could promote cancer cell proliferation, and which were enriched in cell cycle pathways in GSEA analysis for CCA. Therefore, these circRNA-miRNA-mRNA networks probably play significant roles in regulating the cell cycle.

Notably, *ZWINT* was overexpressed in multiple tumors such as HCC [34], prostate, and lung cancer [35], and it was associated with poor survival through upregulating cell-cycle proteins. A high level of *NEK2* was also associated with a poor prognosis in pancreatic cancer [36]. In HCC, *NEK2* activated the PP1/Akt, Wnt, and mitogen-activated protein kinase pathways [37], thereby exerting a carcinogenic function. Thus, our results outline the potential circRNAs-related signaling pathways such as hsa_circRNA_0000673/miR-548b-3p/*CCNB1* and hsa_circRNA_0060144/miR-616-3p/*NEK2*.

There are several limitations to this study. First, mechanistic studies are needed to verify the molecular functions of the five DE circRNAs. Second, due to a lack of following-up, survival analyses were not performed on hsa_circ_0000673. Third, a high false-positive was inevitable due to the sample size of the microarray and qRT-PCR. These limitations indicate that more clinical data and dCCA cases are needed to complete analyses for more convincing conclusions. Systematic molecular biology experiments should be carried out to verify the potential signaling pathways presented in the current study.

## CONCLUSIONS

In summary, we first obtained a comprehensive circRNA profile in dCCA and identified 171 dysregulated circRNAs. Three up- and two downregulated circRNAs were validated with qRT-PCR. A circRNA-miRNA-mRNA network was constructed, and which may regulate the cell cycle in dCCA. The expression of hsa_circRNA_0000673 was upregulated in dCCA tissues and associated with tumor progression. *In vitro* experiments indicate that hsa_circRNA_0000673 may function as an oncogene and serve as a potential biomarker and a therapeutic target for dCCA treatment.

## MATERIALS AND METHODS

### Patients and samples

From January to December 2019, 40 patients diagnosed with dCCA who subsequently underwent pancreaticoduodenectomy were enrolled in the current study, at the Department of Hepatobiliary Surgery in Beijing Chao-Yang Hospital, Capital Medical University (Beijing, China). The diagnosis was confirmed with pathology results. Adjuvant radiotherapy and chemotherapy were not performed preoperatively. Paired tumor and adjacent healthy fresh tissues were immediately frozen in liquid nitrogen for two hours, then stored at -80° C. Among the 40 pairs of dCCA tissues, six pairs were selected for circRNA microarray analysis, and all the tissue samples (including the samples for microarray) were used for qRT-PCR validation. All the patients signed the informed consent form, and the study was approved by the hospital ethics committee.

### Cell culture and transfection

The human CCA cell lines RBE, TFK-1, KMBC, and HuH28 were purchased from the American Type Culture Collection (Manassas, VA, USA). Cells were cultured with RPMI-1640 medium (Hyclone, Logan, UT, USA) supplemented with 10% FBS and penicillin/streptomycin at 37° C with 5% CO_2_.

SiRNA against hsa_circ_0000673 (si-circRNA: 5′-TATTAGACGTTCTTGGTGAAAATCTTTACA-3′) was designed and synthesized by GenePharma (Shanghai, China). Following the manufacturer’s instructions, 50 nM of siRNA was transfected into RBE and KMBC cells using Lipofectamine 3000 reagent (Invitrogen, Carlsbad, CA, USA).

### Circular RNA microarray detection and differential expression analyses

The paired tumor and adjacent normal tissue samples from six dCCA patients were used for microarray detection. Total RNA from each sample was extracted with TRIzol reagent (Invitrogen) and quantified using the NanoDrop ND-1000 (Thermo Fisher, USA). The products were digested with Rnase R (Epicentre, Madison, WI, USA), and the linear RNAs were removed. The enriched circRNAs were amplified and transcribed into fluorescent cRNA utilizing a random primer method (Super RNA Labeling Kit, Arraystar, Rockville, MD, USA). Labeled cRNAs were hybridized onto the Arraystar Human circRNA Array V2 (8x15K) and incubated for 17 h at 65° C in a hybridization oven (Agilent, Santa Clara, CA, USA). After washing the slides, the arrays were scanned using an Agilent G2505C scanner.

Agilent Feature Extraction software (version 11.0.1.1) was used to analyze acquired array images. Quantile normalization of the raw data was performed using R software. CircRNA samples with at least three out of six having flags in “P” or “M” (“All Targets Value”) were retained for further analyses. The limma R package was applied to identify DE circRNAs between tumor and normal tissues (|log_2_FC| > 1 and *p* < 0.05). We used pheatmap and Rcircos R packages to visualize the DE circRNAs and their distributions in human chromosomes.

### GO and KEGG analyses for differentially expressed circular RNAs

To further understand the function of DE circRNAs, the clusterProfiler R package was used to perform GO and KEGG pathway enrichment analyses for host genes of DE circRNAs [38]. The results were visualized with the ggplot2 R package [39].

### Quantitative real-time polymerase chain reaction

Total RNAs from cells and tissue samples were isolated with TRIzol reagent (Invitrogen) and transcribed into the first-strand cDNA using rtStar™ First-Strand cDNA Synthesis Kit (Arraystar Inc.). Specific primers were designed with Primer 5.0 and synthesized by Yingjun Biotechnology Co., Ltd (Shanghai, China). Forward and reverse primers were located at the 3’ and 5’ ends of circRNA, respectively, which ensured that the amplified sequence included the back-splice junction site of circRNAs (the sequences are shown in Supplementary Table 2). Following the manufacturer’s instructions, qRT-PCR was performed with Arraystar SYBR® Green Real-time qPCR Master Mix (Arraystar Inc.). The expression of circRNA was analyzed using the 2^-ΔΔCt^ method and normalized to β-actin expression levels.

### Identification of a prognostic biomarker for distal cholangiocarcinoma

To evaluate whether the expression values of circRNAs could distinguish the tumor from normal tissue samples, ROC curves were applied, and AUCs were calculated. Meanwhile, clinicopathological variables of dCCA patients including tumor size, regional lymph node metastasis, distant metastasis, TNM stage grouping, histological grade, and resection status were extracted and classified according to the 8^th^ edition of the AJCC. To assess the relationship between hsa_circ_0000673 level and these variables, the patients were divided into high and low-level groups according to the median value of this circRNA. Then the percentages of T1-T2 versus T3-T4, N0-N1 versus N2, negative versus positive metastasis, TNM stage I-II versus stage III-IV, well to moderate versus poor histologic grades, and R0 versus R1 were calculated and compared between the two groups with Fisher’s exact tests.

### Competing miRNA prediction

The potential target miRNAs of the five DE circRNAs were screened out with CSCD (http://gb.whu.edu.cn/CSCD/) and CircInteractome database (https://circinteractome.nia.nih.gov/index.html). An intersection analysis between the results of the two datasets was then performed to identify the possible competing miRNAs.

### Luciferase reporter assay

The wild-type (WT) and mutant (MUT) sequences of five DE circRNAs containing miRNA binding sites were cloned and inserted into the pmirGLO Dual-Luciferase vectors (Promega, Madison, WI, USA), respectively. HEK 293T cells were co-transfected with 50 nM miRNA mimics or negative control and 500 ng/ml wild-type or mutated pmirGLO-circRNA with Lipofectamine 2000 (Invitrogen). After 48 hours, the luciferase activity of each group was measured by the Dual-Luciferase Reporter Assay System (Promega, Madison, WI, USA) following the manufacture’s instruction. All assays were performed in triplicate.

### KEGG pathway enrichment for miRNAs

To illustrate the functions of the target miRNAs of the five DE circRNAs, DIANA-miRPath V2.0 was used to find and visualize the enriched KEGG pathways.

### Construction of a circRNA-miRNA-mRNA network

The putative target mRNAs of miRNAs were obtained by using the miRWalk2.0 database (http://mirwalk.umm.uni-heidelberg.de). We extracted the mRNA expression profile of the CCA cohort from Broad TCGA GDAC (http://gdac.broadinstitute.org), which included 36 tumorous and nine normal samples. A Pearson correlation analysis between miRNA and mRNA were performed, and the significantly negatively correlated (*p* < 0.05) mRNAs were regarded as the target genes of miRNAs.

A PPI network was obtained using the STRING database (https://string-db.org) [40] and visualized with Cytoscape software. A combined score ranging from 0 (low) to 1 (high) represents the correlation of each PPI relationship pair. In the current study, we used an interaction score > 0.4 (moderate) as the cut-off criterion. The MCODE plug-in in Cytoscape software was used to identify the hub genes of the PPI network [41]. The screening conditions were set as degree cut-off = 2, K-Core = 2, and Node Score Cutoff = 0.2. Finally, a circRNA-miRNA-mRNA regulating network in dCCA was constructed. Besides, we verified the expression of hub genes using the mRNA data of GSE32879 [42].

### Cell viability assay

Cell proliferation ability was measured using a CCK-8 assay (Sigma-Aldrich) according to the manufacturer’s instructions. Approximately 2 × 10^3^ cells per well were incubated in 96-well plates. At 0, 24, 48, 72, and 96 h, CCK-8 solution (10μl) was added to each well, and the cells were incubated at 37° C for two h. The optical density (OD) at 450 nm was determined with a SpectraMax microtiter plate reader (Molecular Devices, Carlsbad, CA, USA). Cells in each group were tested in three replicates.

### Transwell assay

Cell migration and invasion assays were performed using a transwell chamber (8 μ pore size; Corning Incorporated, Corning, NY, USA) following the manufacturer’s instructions. Polycarbonate (PC) membrane (Corning) was pre-coated with or without matrigel (Corning) for invasion or migration assays. At 24 h after transfection, 2 × 10^4^ cells in 200 μl serum-free DMEM were seeded into the upper compartment. The lower chamber was filled with 700 μl 20% FBS. After 24 h incubation, the cells located in the upper chamber were erased, and the cells in the lower chamber were fixed with 4% paraformaldehyde for 15 min and stained with 0.1% crystal violet for 15 min (Beyotime Institute of Biotechnology, Jiangsu, China). The stained cells were photographed and counted in five fields with a light microscope.

### Colony formation assay

After 24 h transfection, cells were seeded into 6-well plates and incubated for 14 days. After that, the cells were fixed with 4% paraformaldehyde for 15 min and stained with 0.1% crystal violet for 15 min. The cell colonies were photographed and counted with a light microscope.

### Cell scratch wound assay

In brief, 5 × 10^7^ cells per well were seeded in a 24-well plate and cultured overnight. After that, these cells were scratched with a 200-μl pipette tip to create a wound and washed twice with phosphate-buffered saline (PBS). The cells were photographed at 0, 24, and 48 h, and the width of the wounds were subsequently measured. The wound width in each group was tested in three replicates.

### Statistical analyses

R software version 3.6.0 was used to integrate and analyze the data. A paired sample t-test was used to compare continuous variables between two groups. Continuous variables were shown as the mean ± standard deviation. Fisher’s exact test was applied to compare the categorical variables among groups. The figures were produced using GraphPad Prism 8.0 (GraphPad Software, Inc. La Jolla, CA, USA). A *p*-value < 0.05 was considered statistically significant.

## Supplementary Material

Supplementary Figures

Supplementary Tables
