# Supplementary material for: Comprehensive circular RNA expression profiling constructs a ceRNA network and identifies hsa_circ_0000673 as a novel oncogene in distal cholangiocarcinoma
Source: Aging (Albany NY). 2020 Nov 18;12(22):23251–74. doi: 10.18632/aging.104099 (PMC7746367; doi:10.18632/aging.104099)
Supplement: Supplementary Figures [file aging-12-104099-s001..pdf]

SUPPLEMENTARY FIGURES

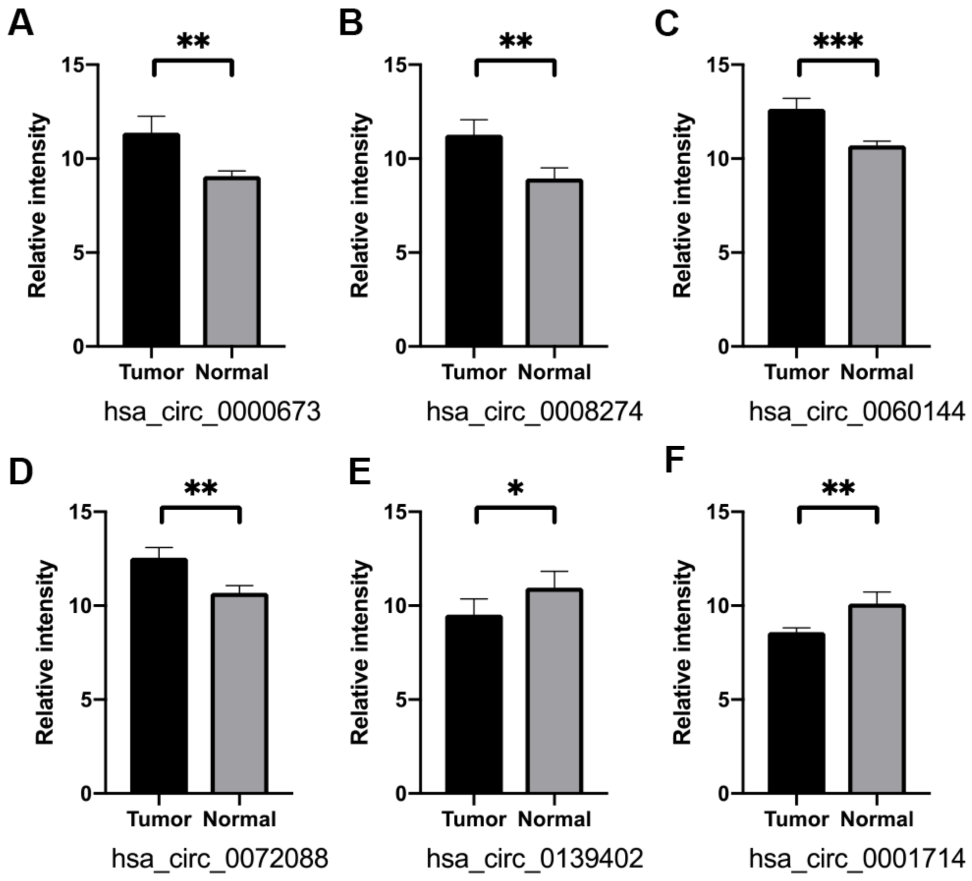

Supplementary Figure 1. Relative intensity of six differentially expressed circular (circ)RNAs by microarray detection. Four significantly upregulated circRNAs, including hsa_circ_0000673, hsa_circ_0008274, hsa_circ_0060144, and hsa_circ_0072088 (**A**–**D**), and two significantly downregulated circRNAs, including hsa_circ_0139402 and hsa_circ_0001714 (**E** and **F**) were identified. $*p < 0.05$, $**p < 0.01$, $***p < 0.001$.

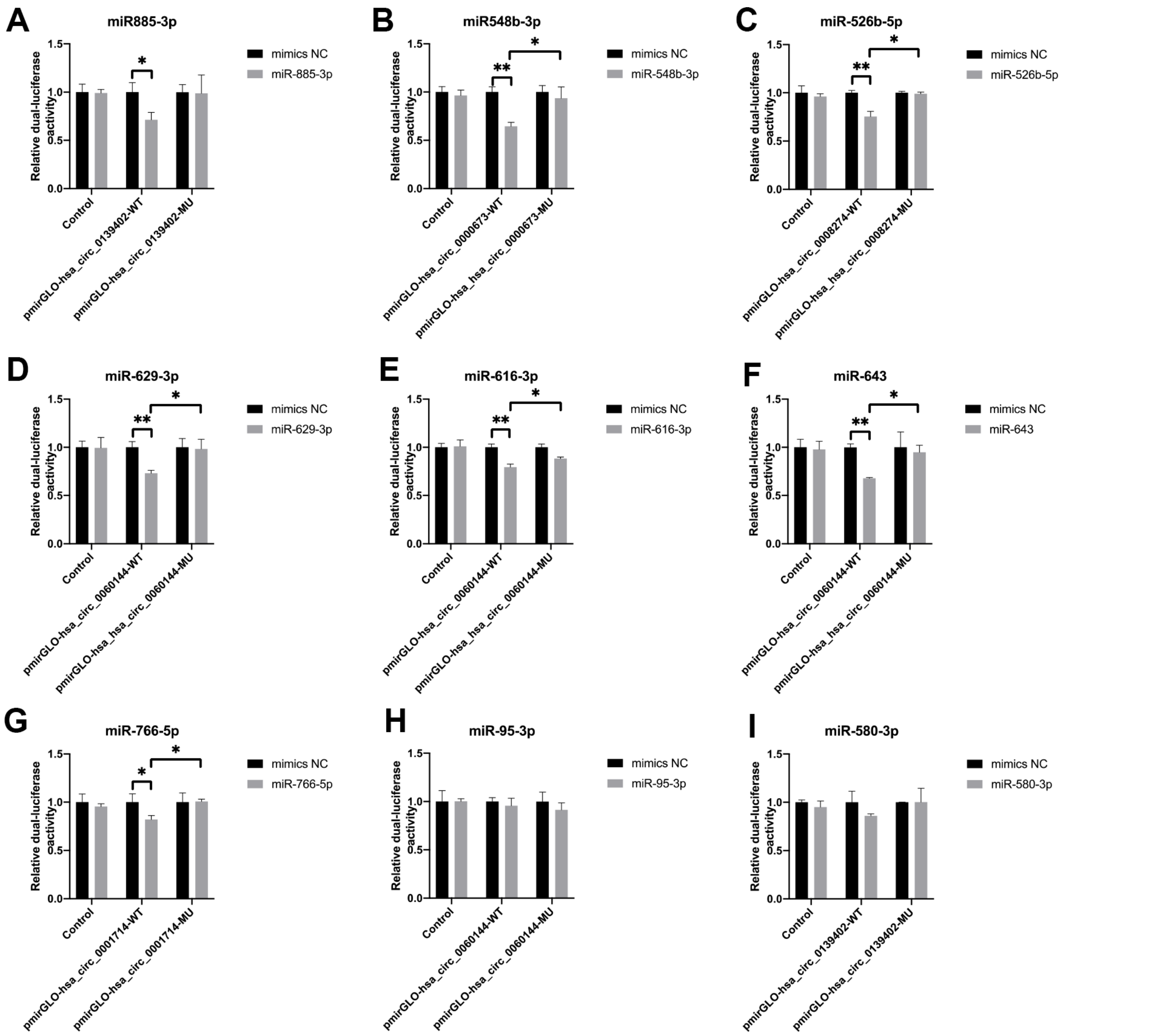

Supplementary Figure 2. Luciferase reporter assays. Seven circular RNA-microRNA binding pairs were proved including hsa_circ_0139402/miR-885-3p (**A**), hsa_circ_0000673/miR-548b-3p (**B**), hsa_circ_0008274/ miR-526b-5p (**C**), hsa_circ_0060144/ (miR- 629-3p, miR-616-3p, and miR-643; **D**–**F**), and hsa_circ_0001714/miR-766-5p (**G**). However, the relationships of the other two pairs, hsa_circ_0060144/miR-95-3p (**H**) and hsa_circ_0139402/miR-580-3p (**I**), were not significant. *p < 0.05, **p < 0.01.

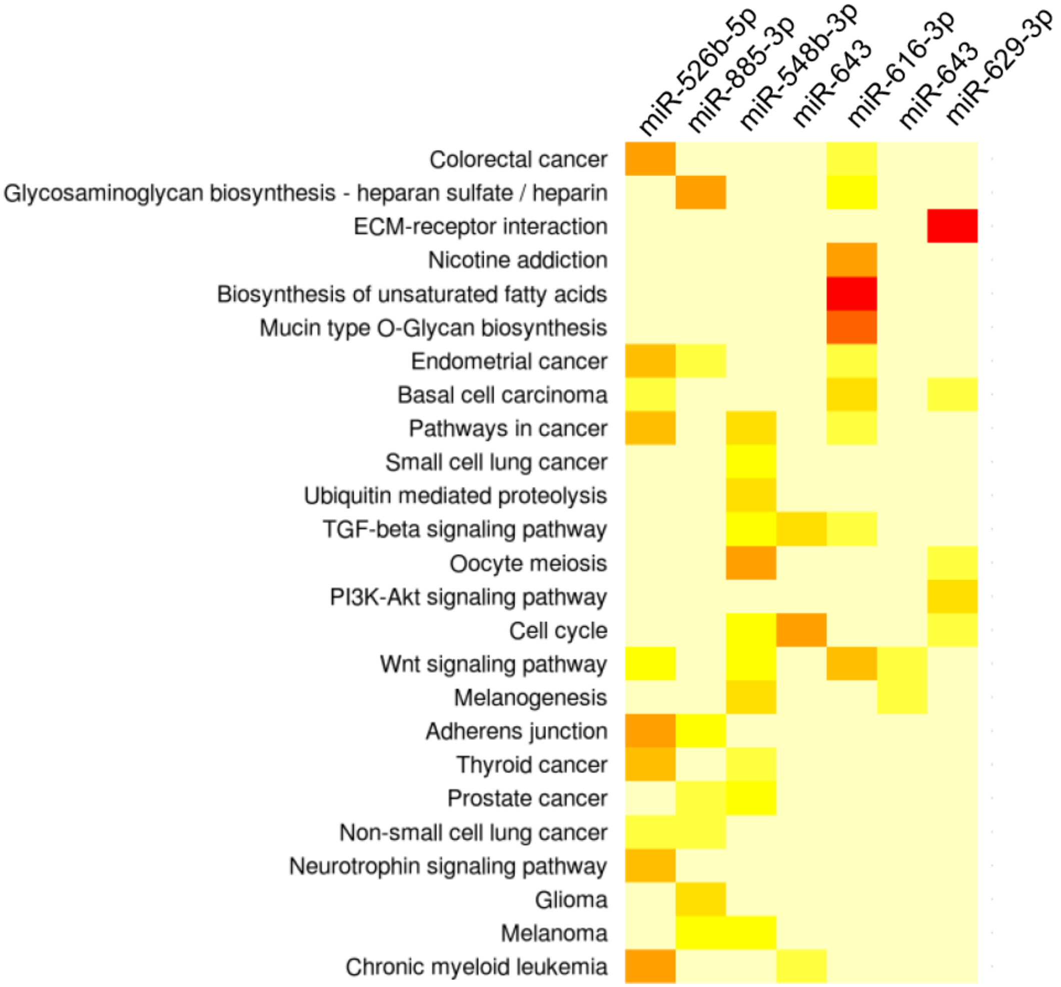

Supplementary Figure 3. KEGG analyses of seven microRNAs corresponding to five differentially expressed circular RNAs. Abbreviation: KEGG: Kyoto Encyclopedia of Gene and Genomes.

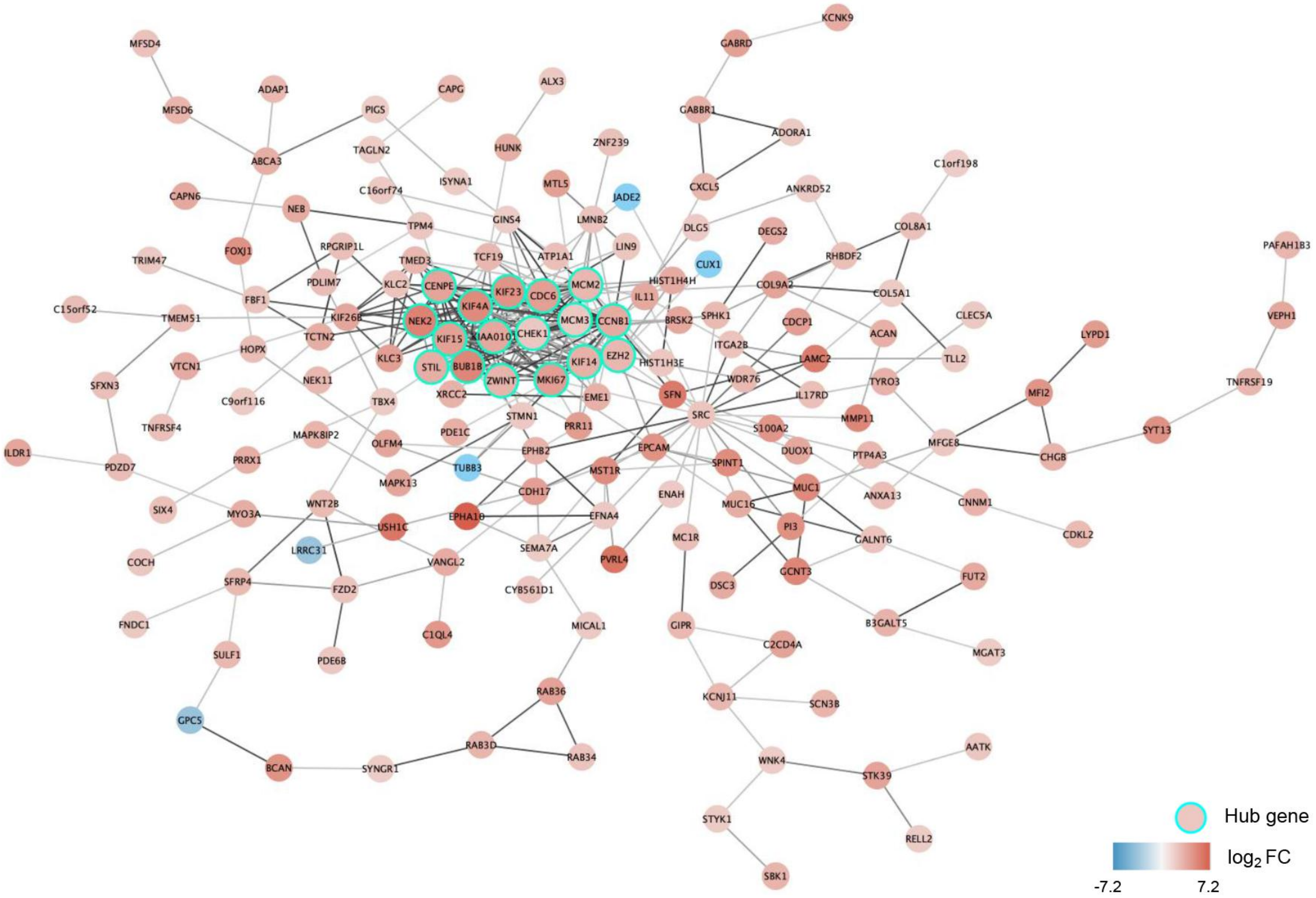

Supplementary Figure 4. Protein-protein interaction (PPI) network and hub genes of cholangiocarcinoma. This network includes 260 target genes and 17 hub genes corresponding to seven microRNAs. The node color changes from blue to red, presenting the fold change of mRNA expression value (tumor versus normal).

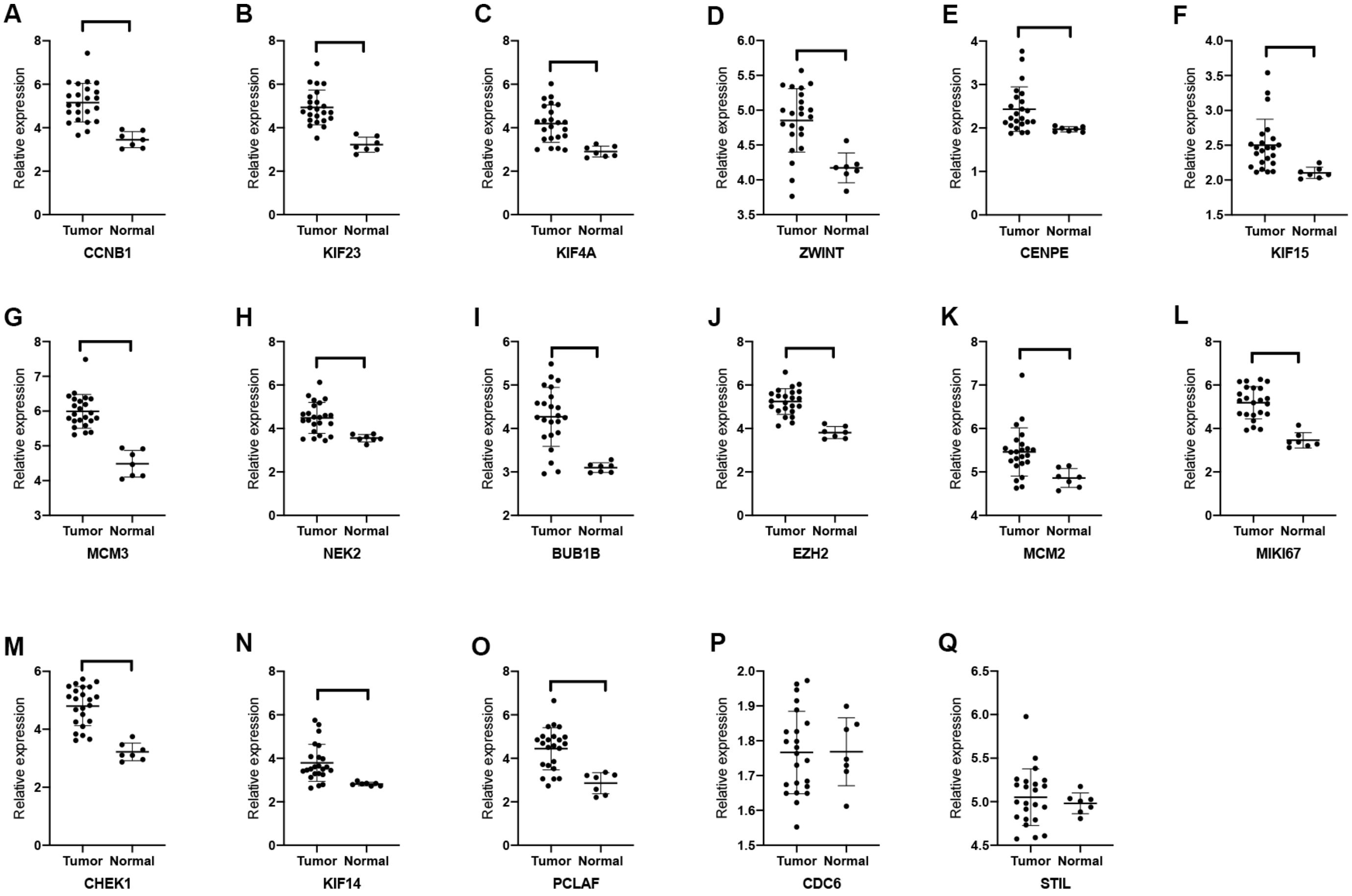

Supplementary Figure 5. Validation of hub genes expression in cholangiocarcinoma (CCA). Among the 17 hub genes, 15 upregulated genes, including CCNB1, KIF23, KIF4A, ZWINT, CENPE, KIF15, MCM3, NEK2, BUB1B, EZH2, MCM2, MIKI67, CHEK1, KIF14, and PCLAF were validated with GSE32879, which contains 23 CCA and seven normal tissues (**A**–**O**). The expression of CDC6 (**P**) and STIL (**Q**) is not different between tumor and healthy tissues. *p < 0.05, **p < 0.01, ***p < 0.001.

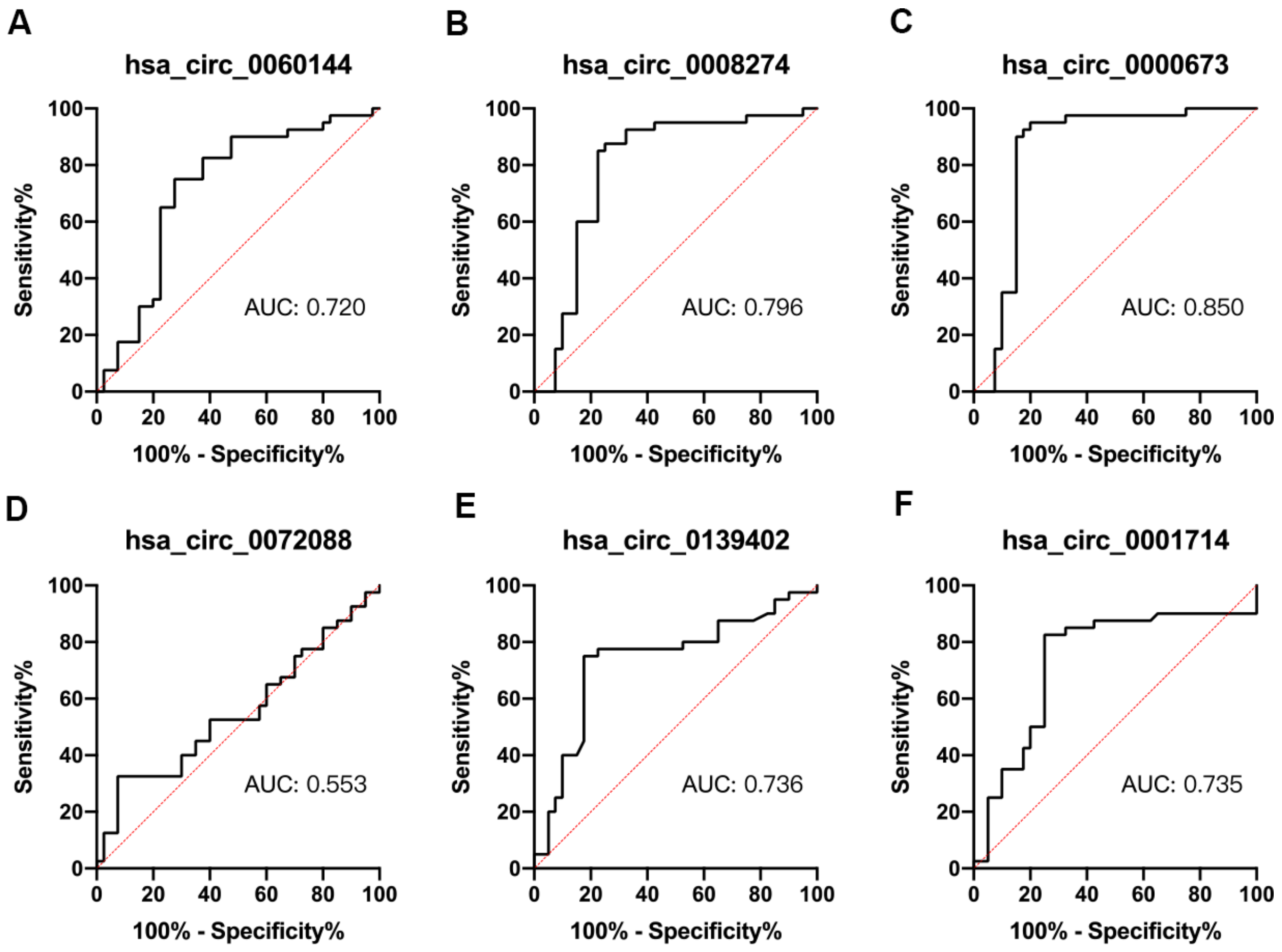

Supplementary Figure 6. Receiver operating characteristic curves of six circular RNAs, including hsa_circ_0060144 (**A**), hsa_circ_0008274 (**B**), hsa_circ_0000673 (**C**), hsa_circ_0072088 (**D**), hsa_circ_0139402 (**E**), and hsa_circ_0001714 (**F**), in distinguishing distal cholangiocarcinoma from healthy samples.
