# Supplementary material for: Comprehensive circular RNA expression profiling constructs a ceRNA network and identifies hsa_circ_0000673 as a novel oncogene in distal cholangiocarcinoma
Source: Aging (Albany NY). 2020 Nov 18;12(22):23251–74. doi: 10.18632/aging.104099 (PMC7746367; doi:10.18632/aging.104099)
Supplement: Supplementary Tables [file aging-12-104099-s002..pdf]

SUPPLEMENTARY TABLES

Supplementary Table 1. Characteristics of the top 10 up- and downregulated circular (circ)RNAs.

circRNA	Alias	circRNA type	log_2 fold change	P.Value	FDR	Chromosome	Strand	Start	End	Host Gene	Regulation
hsa_circRNA_050649	hsa_circ_0050649	exonic	2.50	0.0005	0.10	chr19	-	36245469	36247930	HSPB6	up
hsa_circRNA_406083	hsa_circ_0114695	intronic	2.43	0.0021	0.12	chr20	-	13532533	13581264	TASP1	up
hsa_circRNA_101287	hsa_circ_0008274	exonic	2.32	0.0053	0.16	chr13	-	96485180	96489456	UGGT2	up
hsa_circRNA_101707	hsa_circ_0000673	exonic	2.32	0.0021	0.12	chr16	-	11940357	11940700	RSL1D1	up
hsa_circRNA_101744	hsa_circ_0005699	exonic	2.23	0.0003	0.10	chr16	+	19627435	19663412	C16orf62	up
hsa_circRNA_050648	hsa_circ_0050648	exonic	2.22	0.0003	0.10	chr19	-	36245469	36246795	HSPB6	up
hsa_circRNA_027446	hsa_circ_0027446	exonic	2.20	0.0049	0.15	chr12	+	66221780	66232349	HMGA2	up
hsa_circRNA_025460	hsa_circ_0025460	exonic	2.18	0.0290	0.28	chr12	-	10865809	10868382	YBX3	up
hsa_circRNA_092446	hsa_circ_0000803	intronic	2.16	0.0061	0.16	chr17	-	73775150	73775265	H3F3B	up
hsa_circRNA_404959	hsa_circ_0099201	exonic	2.15	0.0024	0.13	chr12	+	7301567	7303659	CLSTN3	up
hsa_circRNA_038651	hsa_circ_0038651	exonic	3.13	0.0095	0.19	chr16	+	24046739	24046868	PRKCB	down
hsa_circRNA_001226	hsa_circ_0001226	antisense	2.42	0.0065	0.16	chr22	+	36688187	36689461	MYH9	down
hsa_circRNA_016346	hsa_circ_0016346	exonic	2.27	0.0021	0.12	chr1	-	210970849	211192598	KCNH1	down
hsa_circRNA_102700	hsa_circ_0000994	exonic	2.20	0.0487	0.34	chr2	-	40655612	40657444	SLC8A1	down
hsa_circRNA_005232	hsa_circ_0005232	exonic	2.19	0.0457	0.34	chr2	-	40655612	40657441	SLC8A1	down
hsa_circRNA_010553	hsa_circ_0010553	exonic	2.00	0.0048	0.15	chr1	-	22151196	22199917	HSPG2	down
hsa_circRNA_404987	hsa_circ_0098305	sense overlapping	1.79	0.0201	0.26	chr12	-	29492783	29494151	ERGIC2	down
hsa_circRNA_001714	hsa_circ_0001714	exonic	1.53	0.0013	0.11	chr7	-	72861593	72884813	BAZ1B	down
hsa_circRNA_407202	hsa_circ_0139402	exonic	1.43	0.0183	0.25	chr9	-	98231032	98232213	PTCH1	down
hsa_circRNA_103384	hsa_circ_0065898	exonic	1.34	0.0026	0.13	chr3	-	51454240	51456330	VPRBP	down

Supplementary Table 2. Primers of real-time quantitative polymerase chain reaction.

Gene	Primer (5′→3′)
β-actin	F:5'GTGGCCGAGGACTTTGATTG3'<br>R:5'CCTGTAACAACGCATCTCATATT3'
hsa_circ_0000673	F:5'TCTGTAAACCTTCTGTCCAAGA3'<br>R:5'TTTATATTCCTTCTTTAGAGTTTGG3'
hsa_circ_0008274	F:5'CTGCCAGAATTGTCCCAGAA3'<br>R:5'CCACAGCCAGTCTTGAGGAA3'
hsa_circ_0060144	F:5'TGTGGAATTACAAGAGATTTCGA3'<br>R:5'TTTGTCATTTTCAGTCATCAACG3'
has_circ_0072088	F:5'GGCTTGCGGTATCCACTGTCT3'<br>R:5'TCCTCATGGCTGCAATTTTCC3'
hsa_circ_0139402	F:5'GCACTATGCTCCTTTCCTCTTG3'<br>R:5'CTGACGCAGGGGCTTGTA3'
hsa_circ_0001714	F:5'GATTATGAGGTGGCTGGTTTGC3'<br>R:5'GCTGCTTTGTGCTTCCGTATTC3'

F: forward primer, R: reverse primer.
